# Comprehensive analysis of the HIV/AIDS policy-making process in Iran

**DOI:** 10.1186/s12961-019-0466-6

**Published:** 2019-07-19

**Authors:** Rahim Khodayari-Zarnaq, Ali Mohammad Mosaddeghrad, Haidar Nadrian, Neda Kabiri, Hamid Ravaghi

**Affiliations:** 1 Iranian Center of Excellence in Health Management, School of Management and Medical Informatics, Tabriz University of Medical Sciences, Tabriz, Iran; 2 Department of Health Management and Economics, School of Public Health, Tehran University of Medical Sciences, Tehran, Iran; 3 Social Determinants of Health Research Center, Tabriz University of Medical Sciences, Tabriz, Iran; 4 Department of Health Services Management, Iran University of medical sciences, Tehran, Iran

**Keywords:** HIV/AIDS, policy analysis, policy-making, framework analysis

## Abstract

**Background:**

A huge number of people living with HIV*/*AIDS lives in developing countries. Thus, strengthening health systems in these countries is a prerequisite for improving disease prevention and care. After three decades of HIV/AIDS policy-making in Iran, conducting a comprehensive analysis on the policy process seems to be essential. In the present study, we aimed to analyse the HIV/AIDS policy-making process in Iran from 1986 to 2016.

**Methods:**

This was a theory-based, multi-method and retrospective study. Interviewing of key informants and review of policy documents were concurrently conducted to identify and include further key informants (39 participants) and documents in the study. Framework analysis was used to analyse data.

**Results:**

The mean age of participants working in HIV/AIDS policy-making was of 48 years and participants had a mean of 14 years of working experience. Findings were categorized as contextual factors, content of HIV/AIDS policies, actors involved in the policy process, and evidence use in the policy process. Contextual effective factors on the HIV/AIDS policy-making process were categorized into five major themes, namely situational factors, structural-managerial factors, socioeconomic factors, political and legal factors, and international factors. The HIV/AIDS phenomenon in Iran was identified to be deeply rooted in the culture and traditions of society. The HIV/AIDS policy content has, recently, been crystallized in the national strategic plans and harm reduction policies of the country. The policy process has been conducted with a solely governmental top-down approach and is now suffering from poor evidence and lack of sufficient consideration of contextual factors.

**Conclusions:**

There is a great need for change in the approach of government towards the issue and to increase the participation of non-governmental sectors and civil society in the policy process.

## Background

Until recently, Iran was classified among the countries with a low prevalence of HIV/AIDS [1]. However, the emergence of a large HIV epidemic has been recently reported in the literature [2]. According to the latest Iranian official statistics, a total of 30,183 people with HIV were identified at the end of 2015 [3]. Based on the estimations reported by the Ministry of Health (MOH) and international organizations, 80,000 to 120,000 people are living with HIV (PLHIV) in Iran [4]. In recent years, there has been a decrease in the prevalence rate of HIV among three key populations of people who inject drugs, female sex workers and prisoners [5]. A current study in Tehran has also presented a 1.9% prevalence rate of HIV among transgender women [6]. Further, the prevalence rate of HIV among working and street children in Tehran was reported to be 4.5% in 2016 [7]. Like Iran, in many developing countries, HIV/AIDS is considered to a public health concern.

Approximately 90% of PLHIV live in developing countries [8], which urges the need for HIV/AIDS policy development and analysis. With a better understanding on the policy processes, health policy-makers may design effective and appropriate processes and health policy actors may also engage with these processes [9]. Health policy researchers in several previous studies [10–13] have tried to analyse and evaluate HIV/AIDS policies and programmes. In Uganda, a review of AIDS policy development, implementation and evaluation processes was conducted. The main gaps were reported to be limited to indigenous funding to support adherence to anticipated timelines and lack of protocols/standard operating procedures for the processes [12]. In Kenya, after a national policy review of health facility surveys in urban and rural areas, Cawley et al. [14] concluded that there was a wide implementation of the policies promoting access to treatment and retention in care, and a partial or limited implementation of several policies that promote access to HIV testing.

In Iran, in response to the HIV/AIDS epidemic, health policy activists established the AIDS Supreme Council 1 year after the diagnosis of the first cases [15], implemented harm reduction policies [16], and set up drop-in centres and triangular clinics to identify and supervise PLHIV [17]. The council also developed and implemented four National Strategic Plans (NSPs) to control the disease [18]. An important task considered in the fifth development plan of Iran was preventing and controlling the disease and its risk factors [19]. The Iranian government has also committed to collaborate with the international organizations responsible for HIV/AIDS control. In this regard, the government has provided four reports on monitoring the commitment declaration adopted from special meetings of the United Nations General Assembly on AIDS (UNGASS) in 2014 [20, 21].

Now, after three decades of HIV/AIDS policy-making, planning and executive activities in Iran, the conducting of a comprehensive analysis of the policy-making process seems to be essential. Due to lack of HIV/AIDS control [17, 22, 23], the prevalence of HIV/AIDS has been steadily rising in Iran [24, 25]. Challenges such as limited access to high-risk groups due to legal and cultural restrictions and social stigma as well as the high prevalence of risky behaviours among these groups may be mentioned as reasons for failure in the proper control of HIV/AIDS within the country [17, 22, 23]. Moreover, public awareness and knowledge on HIV/AIDS transmission and prevention is relatively low and worrying [26–28]. All these challenges bear the question of why there has been failure in controlling the issue, and whether such a failure is associated to the lack of HIV/AIDS planning and policy-making.

Following a review of the literature we found little evidence on HIV/AIDS policy analysis in Iran. To the best of our knowledge, no study has yet investigated the HIV/AIDS policy-making process in Iran. As defined by Buse et al. [29], a policy-making process is “*the way in which policies are initiated, developed or formulated, negotiated, communicated, implemented and evaluated*”. In the present study, we aimed to analyse the HIV/AIDS policy-making process in Iran from 1986 to 2016, with the hope to provide a clear presentation on the complex relations at play in the process. Our aim was also to obtain a better understanding on how the Iranian public health policy-makers approached the HIV/AIDS issue, as a multi-dimensional phenomenon with a variety of influences. Policy analysis on the issue may be helpful in providing appropriate frameworks with considerations on Iran’s cultural, social and political context as well as the nature of the disease. This study may also help policy-makers in finding a better understanding on the transparency, efficiency and effectiveness of their policy-making process with the hope to develop effective and sustainable HIV/AIDS policies in the future.

## Methods

### Study framework

In this theory-based, multi-method study with conventional content analysis methodology, we conducted a health policy analysis at the national level. The conceptual framework developed by Green et al. [30] was used to study the Iranian HIV/AIDS policy formulation, implementation and evaluation. This conceptual framework is based on a policy triangle [29], in which the four factors of content, context, actors and policy-making process are analysed as the first angle. Thus, as a guide for our study, the policy triangle helped us in developing the following questions: (1) How are the Iranian HIV/AIDS policies made (process)? (2) Who made these policies (actors)? (3) What wider issues may affect the policies (context)? (4) What may be considered as the policy outputs (contents)? We applied this framework to design the study, conduct the interviews and develop the checklist guide for document review and data analysis.

### Study setting

This study was designed and implemented in Tehran, Iran, at the national level, from January 2013 to October 2016. Although the level of policy analysis was national, the opinions of key informants working on HIV/AIDS programmes at the local levels were also investigated.

### Data collection

We initiated the study with a review on the most prominent HIV/AIDS documents, to identify both the core key informants and the other important documents.

#### Policy documents

A comprehensive search on the internet was conducted to select the websites of relevant government institutions, international and national NGOs, health professional councils and associations, as well as religious medical bureaus and research networks. We used the search engine Google to locate such websites, which we then navigated by the tabs and menus available on the homepage (such as policy documents and guidelines, e-library, resources, publications, legislation). In addition, we searched Google Scholar using the following keywords in various combinations with Boolean operators (‘and’, ‘or’): agenda, health policy, health system, guidelines, strategies, plans, and reports. We checked the reference lists of the documents found to expand the list of included documents. Importantly, we used the websites as an entry point to other repositories for national policy documents. More details on this process are previously published [15].

To minimise selection bias, two independent reviewers (BM and RB) screened all documents and selected those that were appropriate for our research question. Our selection involved the use of a pre-determined inclusion and exclusion criteria. We included all agenda-specific published documents relevant to health policy and systems produced between 2000 and 2015.

Where possible, a hardcopy of identified documents was obtained from relevant centres. Some other documents were also identified and obtained through a purposeful approach to the content of documents and to the guidance of key informants during the interviews. A total of 48 documents, including upstream documents (international, national and important reports of country), circulars, regulations, guidelines, provisions approved by the government as well as the papers and important research projects and prevention documents related to the HIV/AIDS policy content in Iran were studied. We had access to the policy documents after providing approval from the centres where these documents were developed. In order to review the documents, a review topic guide was developed based on the issues reported in UNAIDS 2015 progress reports submitted by Iran, Pakistan, Tanzania, Vietnam, Ukraine, and Brazil [31]. To answer the questions in the topic guide, the first author reviewed the documents one by one, and extracted quotations as answers for the questions. The quotations were then imported into MAXQDA v.10 (2011) for data management and analysis.

#### Key informants

We used two methods to identify and to interview key informants. Purposive sampling was used to identify informants in the key roles across sectors relevant to HIV/AIDS policy process. During initial reviews on HIV/AIDS documents, the first three key informants were identified and recruited based on their expertise and their position within the Iranian Research Center for HIV/AIDS (IRCHA). Further informants were recruited through snowball sampling, which involved asking the interviewees to nominate other people they knew with knowledge and experience relevant to the HIV/AIDS policy process. The informants were from different levels of the Iranian health system, including primary, secondary and tertiary health services, intermediate and long-term care services, health policy-makers, healthcare managers, HIV/AIDS clinical practitioners, health education and promotion officers, and faculty members in medical universities. Further key informants and documents were identified and included in the study during interviews and policy document reviews, which were conducted concurrently.

All interviews were conducted by the first author, who has a considerable experience in conducting qualitative interviews with health policy-makers. Our objectives were first described to the participants and all participants signed an informed consent form before the interviews. The semi-structured interviews were conducted applying an interview topic guide. The topic guide was provided based on the issues reported in UNAIDS 2015 progress reports submitted by the abovementioned countries [31]. All interviews were conducted face-to-face. The participants were interviewed at their workplace, and all interviews were audiotaped. The average time for interviews was about 1 h. In cases where a participant did not agree to voice recording, interview notes were taken. This process was continued until theoretical saturation of data, when no new code, category or theme emerged in the last two interviews. In total, 39 participants (26 men and 13 women) were interviewed. The participants included 18 governmental or semi-governmental senior and junior managers, a director of an international organization situated in Iran, 4 NGO administrators, 8 specialists in infectious diseases and HIV/AIDS, 3 researchers in the field of disease, a legislator, and a legislative consultant. In order to have an understanding on the level of consonance between the findings from policy-makers and those from HIV/AIDS patients (as beneficiaries), three PLHIV that had the disease for an average of 5 years and inhabited in Tehran (1 man and 2 women) were interviewed. To do so, we referred to a “Positive Club” situated in a hospital in Tehran and invited PLHIV to participate in the study. We then informed them on the aim of study, and those who accepted our invitation (3 out of 7 patients) signed informed consent forms. The patients were interviewed in a private room of the “Positive Club”, and were insured about confidentiality of the data. The mean age of the patients was 27 years. There was no compensation for respondents to participate in the study. None of interviews was repeated. The interviews were transcribed verbatim and, where possible, were sent back to the participants for any additional comment or correction.

### Data analysis

Framework analysis [32] was used to analyse data and extract themes. The descriptive-analytic phase of the framework included the transcription of interviews, recalling important points during interviews and frequent reading and reviewing the transcripts, and policy documents. The analysis phase included classifying and identifying the main categories, and the interpretation phase included the process of coding. Final interpretation was based on the synthesis and interpretation of data. Descriptive coding was done to identify the superficial and structural relationships between data. Cognitive coding was performed to identify the deep and structural relationships throughout the context. Perceptual analysis was conducted to examine the occurrence, presence, frequency and periodicity of concepts. Interaction analysis was done to study the deep relations between the concepts that eventually led to the final synthesis of data. All these phases were also conducted on the policy documents.

The qualitative data analysis software MAXQDA v10 (2011) was applied to organise and to manage the coding process. The transcripts were read and re-read, and then the initial codes were drawn from data. The codes were collated into themes and a coding frame was developed. All interviews and analyses were conducted by the first author, who was in close discussion within the research team. By his preconceptions, he tried to derive the themes. During data analysis, he had the theoretical framework-based research questions (the questions on the process, actors, context and contents of the Iranian HIV/AIDS policies) in mind.

### Quality of research

Credibility, conformability, dependency and transferability were considered as the criteria for data trustworthiness [33, 34]. Firstly, we enrolled the key informants with enough experience in Iranian HIV/AIDS policies in the study. To increase conformability and to resolve the causes of inconsistency within findings, we tried to interview contrastive cases. Moreover, the high level of researcher engagement in all stages of the study and allocation of sufficient time increased the conformability of our study. To account for inter-rater reliability, one in five raw transcripts was randomly selected and coded by a second researcher. Therefore, agreement with the themes was checked and the deduction of similar themes from the texts by both researchers was ensured. No change was made to the emerging themes, but some minor changes were made to the terminology.

In order to increase dependency, we tried to consider and apply all comments noted by the research team members. The results of data analysis were presented to some participants, and their feedback was considered in the data analysis process. To increase transferability, we also tried to include appropriate participants in the study. Data collection and analysis were carried out simultaneously.

## Results

**Fig. 1:**
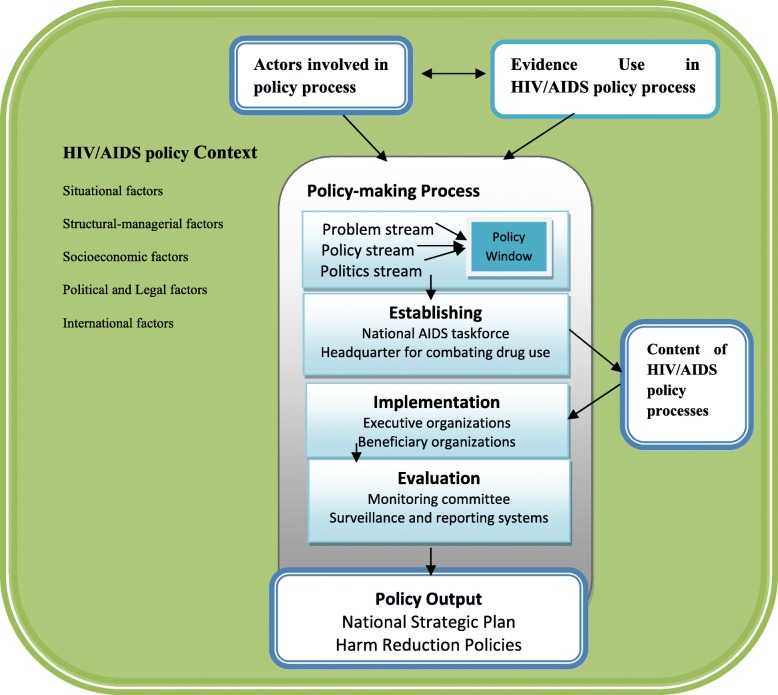
An illustration of how the factors of context, content, actors and evidence intersect to shape the HIV/AIDS policy-making process in Iran

The mean age of participants was 48 years and the mean number years of working experience in HIV/AIDS policy-making was 14. The level of education for a majority of participants was philosophy and general medical doctor. The study findings are presented in four sections, namely contextual factors, content, actors and evidence. Figure 1 illustrates a clear picture of how these factors intersect to shape the HIV/AIDS policy-making process in Iran.

### HIV/AIDS policy context in Iran

Keeping in mind the Lichter approach [35] and based on the opinions of participants in our study, the contextual effective factors on HIV/AIDS policy-making in Iran were categorized into five major themes, namely situational factors, structural-managerial factors, socioeconomic factors, political and legal factors, and international factors. The themes and related sub-themes, as well as some appropriate quotes, are presented in Table 1.

**Table 1:** Contextual factors affecting the HIV/AIDS policy-making process

Themes	Sub-themes
Situational factors	Synthetic and psychoactive drugs “*A problem we have in this area, is the high supply and easy accessibility to drugs and psychoactive substances, which makes the control of substance use difficult*” (participant 15). Child labour and street children “*I, myself, have heard from one of health ministry administrators that the frequency of AIDS among street children is fourfold higher than its mean among the population*” (participant 7). Consumerist lifestyle and irresponsibility “*Within these nine years that I have worked as a consultant, I have seen that the luxury-oriented lifestyle of people and their unrestrained behaviors have resulted in disruption between family relations and … a majority of young people have turned toward wrong sexual behaviors, which, well, have altered the mode of HIV transmission towards the sexual way*” (participant 27). Virtual networks “*Currently, judiciary announced that they have arrested a band who misused virtual networks to advertise illegitimate concubine marriage and earned a huge amount of money…*” (participant 8). Migration to the margins of metropolises “*In recent years, migration to metropolises has increased. Well, they come to the cities to have a good income. However, due to weak financial ability, they inhabit in marginal settings, where there is a prone environment for unemployment, addiction, sex works and…*” (participant 3).
Structural-managerial factors	Inter-sectoral structure “*I attend the different meetings of CCM. The thing I see there is that there is a low level of coherence and discipline between the organizations…*” (participant 18). Intra-sectoral structure “*… I have figured out that the main cause of our problems to prevent/control AIDS in Iran is that the issue has no influential custodian and trustee manager…*” (participant 9). Executive management “*… in many situations, when they* [the representatives of related organisations] *come to the meetings, they do not have a real structure to execute the programmes, and it is difficult for them to define AIDS control programmes within their routine duties*” (participant 8). Financing and resource provision “*… but, this ministry do not have a good budget for such a work. In real, there is not such a budget to be allocated to such works* (AIDS control programmes)” (participant 14).
Socioeconomic factors	Issues related to traditional culture “*AIDS has a social obscenity, which cannot be easily discussed about, informed about or educated on. The reason is the interrelation between AIDS and sexual issues*” (participant 15). Issues related to religion “*When one is going to promote condom use within society, it means that he is going to promote out-of-marriage relationships. Well, this is in contrast to what is said in our religion*” (participant 17). HIV/AIDS and sexuality as a taboo “*As long as there is a taboo on discussing sexual issues, there would not be a good progress in our situation on HIV/AIDS issue…*” (participant 15). Pervasive stigma of HIV/AIDS “*… this stigma is such a horrible stigma that a HIV positive patient refrain from being hospitalized in this ward [infectious diseases ward], which is a ward for all kinds of infectious diseases. Because, they fear that their relatives or friends know about their disease* [AIDS] *…*” (participant 33). “*As soon as they* [people] *know that you are HIV positive, they think that you have had the disease from unconventional way* [sexual way]. *Once I was in a dentistry office when the administrator asked: When did you get? It doesn’t become you, at all! Soon she thought that I have gotten it from that way!*” (participant 38) “*Ordinary people don’t understand us at all! They think that we have committed a big sin, and now we don’t have right to live in this city, like others …*” (participant 36) “*As soon as the dentist heard that I am HIV positive, asked me respectfully to leave the dentistry office as soon as possible …, well, do you expect us to blaze that we have HIV wherever we go?!*” (participant 38) Traditional education system “*We see in our religious books that the rulings on sexual issues are openly described …. But, when one says that such issues should be educated to public, some says that this is in contrast to our religion*” (participant 21). Addiction to drugs “*During these two years that I have worked as a consultant, almost all of people who referred to me have had the disease from sexual way. A majority had also used such drugs* [the new psychoactive substances] *and then had had sexual relations*” (participant 31).
Political and Legal factors	Centralized political system “*AIDS policy-making in the country has highlighted the participating role of private sector in terms of service delivery to increase the level of service accessibility among the patients*” (participant 2). Top-down policy-making “*Some of senior managers are extremely sensitive toward some statistics and indices related to AIDS, and so do not like those statistics to be spoken about*” (participant 21). Lack of trust into NGOs “*In general, the NGOs in our country are considered as a threat, and so are not allowed to be well developed*.” (participant 12). Conflict between macro policies “*Coming the policy of growing birth, the contraceptives are also disbanded, which has become a new dilemma*” (participant 15).
International factors	International agencies “*… through a part of available resources, global fund is influential on the domains of addiction and AIDS*” (participant 34). International trends and events “*The pressure and the influences of international organizations to execute their emphasized policies have resulted in an emphasis on the approaches that are not relevant to the culture and the context of our society*” (participant 17).

The findings on interviews with the PLHIV were mainly around the pervasive stigma of HIV/AIDS. The patients believed that they were not understood by other people who did not have exposure to HIV-positive patients and complained about medical and healthcare personnel. They explained that they are usually stigmatized as sex workers (particularly women), which makes them face negative mental consequences, like humiliation, chronic stress and anxiety, and depression (Table 1).

### Content of HIV/AIDS policies and policy-making processes

**Fig. 2:**
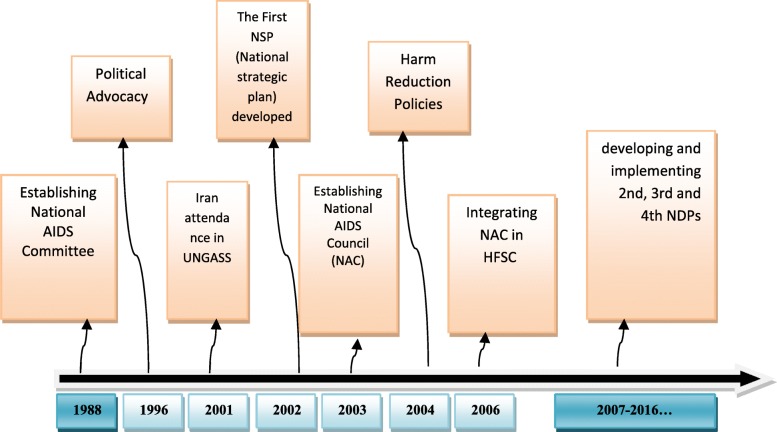
Timeline of the HIV/AIDS policy-making process in Iran, 1988–2016

Figure 2 illustrates the time trend of HIV/AIDS policy-making process in Iran from 1986 to date. The country’s first action following the incidence of the first HIV case (1986) was to form a high-level technical committee in 1988. At the early years, the actions of the committee were mainly passive and concentrated on adopting some policies to provide safe blood. In 1996, following the outbreak of the HIV epidemic among intravenous drug abusers in some prisons, attention was drawn towards this group. However, due to different attitudes toward drug use and the ways they were dealt with by competent authorities, developing and implementing effective policies appeared difficult. Before the outbreak of disease within Iranian prisons, the viewpoints were mainly focused on a society free of high-risk behaviours and, thus, restraining and legally dealing with drug users. During this period, Iran went from the small outbreak stage to a concentrated epidemic stage, which urged officials to begin developing harm reduction programmes. Simultaneously, some other activities took place, including appropriate rallying to win the support of politicians and high-ranking political and religious authorities to facilitate the implementation of harm reduction policies.

The first aid programmes to quit drugs and to develop harm reduction policies were planned in prisons followed by implementation in communities. The harm reduction policies were directed toward the distribution of sterile needles and syringes for single use to avoid common injection (the main transmission pathway of HIV at that time) between the consumers. Provision of methadone treatment for addicts was another harm reduction policy. Subsequently, some policies were put into action, including the distribution of condoms in prisons and communities, the distribution of disposable razors to avoid common use between prisoners, and the provision of religious meeting rooms for prisoners and their wives to have safe sexual relations.

In 2001, Iran participated in UNGASS and, 1 year later, the first HIV*/*AIDS programme was developed. Although the Iranian cabinet did not approve the programme, some efforts were made to implement it within the country. In 2003, the Supreme Council for HIV/AIDS Prevention Planning (SCHAPP) was formed and approved by the cabinet and, accordingly, national and provincial committees were formed as a subset of the council. Three years later, in 2006, SCHAPP was dissolved and integrated into the Health Supreme Council and Food Security, with the previously mentioned committees also becoming its subsets. The dissolution of SCHAPP reduced the effectiveness of its subcommittees so that the majority were semi-active, inactive or passive, except for two subcommittees, namely the Care and Treatment subcommittee, which was responsible for providing and updating the treatment guidelines for sufferers, and the Harm Reduction subcommittee, which operated as a subset for an anti-drug campaign.

In recent years, Iran’s policy content in the area of HIV/AIDS has been crystallized in the national strategic plans. The third strategic plan, which ended in 2015, included specific HIV/AIDS strategies and operational goals approved by the cabinet. This plan indicated the content of Iran’s policies on the disease and emphasized the domains of education and information, harm reduction, prevention from sexual transmission, consultation, care and treatment services, patient support, mother-to-child transmission prevention, and the political and organisational infrastructure. Some quotations are presented below:

“*You see that the index of condom use among the target groups of the programmes is still less than 50%. Different studies have shown this. I believe that with such new policies that are now being applied in the country, such indices will surely get worse*.” (participant 20).

“*Although the pattern of HIV transmission is getting shifted towards sexual relations, in my idea, the injection way is still the most important way. More attention should be paid to harm reduction policies, also, the range of under coverage individuals for such policies should be increased*.” (participant 4).

### Actors involved in policy-making processes

**Fig. 3:**
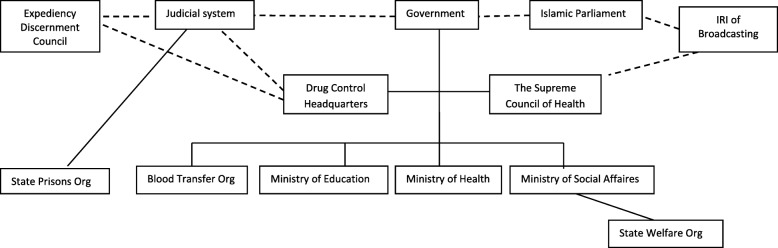
Main actors involved in HIV/AIDS policy-making process in Iran

The multi-sectoral nature of HIV/AIDS prevention and its close relationship with the fields of addiction and social harms urge the need for wide inter-sectoral coordination. The HIV/AIDS Supreme Council was formed to determine the policies of executive agencies, and to coordinate the stakeholders. However, in recent years, the mechanisms of inter-sectoral coordination have actually been weakened after the integration of the SCHAPP into the Health Supreme Council and Food Security. Since the council was in charge of public health with a lot of different agendas, some public health concerns other than HIV/AIDS were given greater priority and were paid more attention by stakeholders and politicians. Some of these public health concerns included the goals and challenges related to the health development plan, the increasing rate of non-communicable diseases like cancer, coronary heart diseases and sedentary lifestyle, as well as food security issues. Figure 3 shows the opinions of main actors in the field of HIV/AIDS in Iran.

Some actors believed that returning to the HIV/AIDS Supreme Council is a solution for the current situation. Some others posed the current challenges in the work of National Committee and suggested the lack of the governmental authority system (the MOH) in giving priority to HIV/AIDS prevention programmes. Some other key informants considered HIV/AIDS prevention policy-making as a complex task and believed there was a great lack in HIV/AIDS policy-making. They believed that a higher-level structure of the government should be involved in the process rather than an executive branch like the MOH. For instance, at least three branches of the government have to be involved into HIV/AIDS policy-making process to assure the necessary authority needed for strategic policy-making. The reason for this high-level integration was announced to be the existence of serious cultural and attitudinal obstacles that may be only resolved by a good level of interaction between the three branches. The following are some quotations:

“*Although the National Strategic Plan is determined by the prime minister, there is not such an executive guarantee for the plan. In my idea, the AIDS office is not fully supported to completely execute the programmes*” (participant 2).

“ *… the first and the second plan* [the national strategic plans] *were only written on sheets and were delivered to the related organizations, and the third plan were presented with the signature of the vice president. But, no money is allocated to a plan, how it can be implemented?!*” (participant 7).

### Use of evidence in HIV/AIDS policy-making process

As the key informants noted, the first problem of HIV/AIDS policy-making in Iran was a lack of evidence in some specific areas (such as information related to homosexuality, or the impact of synthetic drugs on HIV transmission). They believed on a lack of strong, reliable and nationwide evidence in many aspects of the issue, and commented that a majority of the evidence was based on local research in particular places and time periods. Since HIV/AIDS researchers usually pursue their personal interests, appropriate investigations were inadequate. Additionally, minimum budgets were often allocated to HIV/AIDS research. Another problem reported by participants was the lack of person-power and facilities to produce evidence. Managers were reported to be not interested in producing evidence, and thus little evidence was produced from running programmes. This lack of evidence has often resulted in various problems, in a way that using trial and error was usually common in the executive agencies. The associate managers sometimes have to meet the everyday needs of people, and may temporarily set aside the evidence. For example, despite the evidence emphasizing inefficiency and even harmfulness of gathering addicts from streets and transferring them to camps, the Iranian managers still approach the issue in this manner as an effort to respond to people’s urgent requests for a drug-free environment. Sometimes, the political viewpoints or the personal opinions of the managers affect the decision of whether or not to use evidence. Lack of interested and competent management in applying evidence, lack of accountability among the managers, high workload of managers and lack of time to refer to evidence were other managerial factors affecting evidence non-use. However, it should be acknowledged that the evidence relating to some policies, like harm reduction programmes, has been advantageously used in many cases. Moreover, the presence of international organisations, such as UNAIDS and WHO, has improved the use of evidence in HIV/AIDS policy-making in Iran. Here are some quotations from the participants:

“*In the disseminated national HIV/AIDS reports, in some domains, like the sexual and MSM issues, there is no evidence to be cited. It is clear, the lack of evidence will surely deviate the vision and the policies*” (participant 8).

“*In the current national AIDS strategic plan, the cost-effectiveness and cost-benefit analyses are ignored. Well, this results in wrong evaluation and infirm evidence*” (participant 23).

“*Some evidences are approached politically in the higher level of the government, and some other considerations are focused. For example, the politic of country is not to highlight the issue* [that evidence shows].” (participant 15).

## Discussion

### Main outputs of AIDS policies and their challenges

Based on our results, the main outcomes of the HIV/AIDS policy process in Iran were harm reduction policies and an HIV/AIDS national strategic plan, which have had effects on different areas of the issue. Harm reduction programmes in Iran were initially presented in the form of triangular clinics established in the communities and prisons throughout the country. Iran’s efforts in controlling HIV/AIDS in prisons have been known as one of the most successful efforts in the world [36]. Harm reduction policies were often considered among the main programmes of the government, when implementing the national strategic plans. Such policies have also had a good efficacy in similar contexts in other countries [37]. However, the key informants in Iran have always been in doubt about continuing these policies, and have had fear from changing and weakening these programmes [16].

As participants explained, a challenge of implementing harm reduction policies was the failure of Iranian governments in generalizing the programme towards risky sexual behaviours due to difficulties in engaging sufferers into the programme, such as the reluctance of sufferers to join the programme, legal gaps and lack of suitable strategies. In contrast, other countries, like Vietnam, achieved success in controlling HIV/AIDS through similar programmes [37, 38]. Our participants believed that the attitudes of some politicians and managers in Iran have had an impact on the poor development of the programme towards risky sexual behaviours. Despite successful implementation of the programme in Iranian prisons [20, 36], the fear of weakening and/or stopping the programme has even pervaded prisoner health. Participants believed on poor interactions and communications between health professionals and judicial authorities as a weak point for harm reduction policies. On the one hand, health authorities were not able to effectively draw the stakeholders’ attention towards strengthening and expanding the policy. On the other hand, the judicial system as well as some policy-makers and social and educational top managers were sceptical on the policy, and worried about its potential socio-cultural impacts on the society. Such stakeholders preferred those policies that were based on drug use control and criminalization of illicit sexual behaviour and, thus, did not agree with implementing harm reduction policies within the community. This may be noted as an obvious obstacle at the high level of Iranian HIV/AIDS policy-making, which facilitated the spread, rather than control, of HIV [15, 16, 39, 40].

Although the fifth Iranian development plan implementation is over, there is still a superficial understanding on the true nature of the HIV/AIDS programme in Iranian society. As participants reported, the contextual factors have not been well considered and analysed. As a consequence, the total responsibility of HIV/AIDS programme was given to the MOH, which means that HIV/AIDS was considered a health issue rather than a sociocultural concern. Therefore, a challenge in inter-sectoral response was aroused and, eventually, the programme failed to achieve the desired goals. Such an issue was reported in the HIV/AIDS programme of other countries, including South Africa [41]. Our participants believed that HIV/AIDS and its related behaviours was stigma-inducing in the Iranian society, which caused a limited access to high-risk populations. In our study, all participants, including the PLHIV, were in agreement on pervasive stigma of the disease within the country. They believed on the failure of policies related to HIV/AIDS stigma reduction among Iranian communities. They explained the problem to be worse when there was still a high level of stigmatization in attitudes toward patients from clinical practitioners and healthcare providers. Additionally, a general knowledge on HIV/AIDS among the Iranian population was still limited and the attitudes were still negative, sensitive and precautionary [28, 42, 43]. Moreover, the roles of key stakeholders and the effectiveness of their activities have always been in doubt, due to poor coordination and interaction between the stakeholders over the years [44, 45].

One of the main objectives of the NSP was strengthening HIV/AIDS programme infrastructure such as alleviating the legal gap and winning the support of high and influential officials for the policies [46]. However, participants believed that no practical action has been taken to promote the interaction between the stakeholders. Therefore, the policy-making process was considered still far away from being restoring and strengthening.

In terms of the HIV/AIDS policy content, our participants reported a gap between the data and required evidence and, thus, there was no precise plan in the NSP to approach some high-risk groups like men who have sex with men [47]. As participants commented, and based on our document reviews, there was a great lack of evidence for policy-making in HIV/AIDS-related issues such as those related to street women and children. Previous studies also pointed out to the lack of evidence and of an evidence network for HIV/AIDS policy-making in Iran [48, 49]. Many key informants in the present study acknowledged the addiction’s disguise and the growing prevalence of stimulant drugs within the country. However, there was practically no appropriate policy likely due to the lack of evidence.

Finally, a lack of precise HIV/AIDS laws was reported to intensify the confusion in policy-making, administrative systems and non-effective trustees, which may have negatively impacted the role of key stakeholders in the policy-making process. Our participants also reported a lack of interaction between responsible institutions for controlling HIV/AIDS, particularly among high-risk populations. All these problems may cause serious social damage to Iranian society, like those caused by drug addiction.

### Methodological considerations and study limitations

Applying a pre-defined framework for this study was helpful in systematic analysis of the policy process and research. However, there is the fact that some important factors and features may be neglected, which should be considered as a limitation for our study. To overcome this limitation, we maintained a critical view on the codes and a deep contemplation about their nature during data analysis. Although the limitation may not entirely be overcome, this approach was definitely helpful. Moreover, discussing and comparing the results of our study with those of other studies with and without a particular framework provided us with a comprehensive analysis on the HIV/AIDS policy process in Iran.

Considering the significance of context in Iranian HIV/AIDS policy-making and its high impact on the other parts of framework, we chose it as the basis for our study. In summary, the framework by Green et al. [30] was found to be helpful in exploring the process of HIV/AIDS policy-making in the Iranian context. The current literature on HIV/AIDS policy-making was also found to be extremely inadequate, urging the need for further studies.

## Conclusions

Our results demonstrated that the HIV/AIDS policy process in Iran was considered a governmental process alone, and the role of civil society and the NGOs within the process seems to be entirely ignored. The historical and political context of public policy-making in Iran has strengthened such an approach towards HIV/AIDS policy-making. Moreover, several challenges were identified in the policy process, including difficult access to high-risk groups, a low level of population coverage in HIV/AIDS prevention/treatment programmes, and lack of strong and sufficient evidence for policy-making. In order to overcome the challenges, there is a great need for change in the government’s approach towards the issue, and for active participation of non-governmental sectors and civil society in the policy process. Furthermore, the dominance of therapeutic approaches within the health system, and too much emphasis on the aspects of treatment and rehabilitation rather than prevention in both the addiction and HIV/AIDS domains, urge the need for overall reorientation in HIV/AIDS policy-making.

The analysis of HIV/AIDS policy-making process conducted in the present study may be helpful in answering the question of why the HIV/AIDS initial challenges are still present in Iranian society after three decades of the emergence of the disease in the country.

## Data Availability

The datasets used and/or analysed during the current study are available from the corresponding author on reasonable request.
